# Divergent humoral responses to 23-valent pneumococcal polysaccharide vaccine in critically-ill burn and neurosurgical patients

**DOI:** 10.1371/journal.pone.0197037

**Published:** 2018-05-14

**Authors:** Scott W. Mueller, Laura J. Baumgartner, Rob MacLaren, Robert Neumann, Arek J. Wiktor, Tyree H. Kiser, Gordon Lindberg, Luis Cava, Douglas N. Fish, Edward N. Janoff

**Affiliations:** 1 Department of Clinical Pharmacy, University of Colorado Skaggs School of Pharmacy and Pharmaceutical Sciences, Aurora, Colorado, United States of America; 2 Department of Clinical Sciences, Touro University California College of Pharmacy, Vallejo, California, United States of America; 3 Department of Neurosurgery, University of Colorado School of Medicine, Aurora, Colorado, United States of America; 4 Department of Surgery, University of Colorado School of Medicine, Aurora, Colorado United States of America; 5 Division of Infectious Disease, Mucosal and Vaccine Research Program Colorado (MAVRC), University of Colorado School of Medicine and Denver Veterans Affairs Medical Center, Aurora, Colorado, United States of America; University of Colorado Denver, UNITED STATES

## Abstract

**Introduction:**

Critically ill hospitalized patients are at increased risk of infection so we assessed the immunogenicity of 23-valent pneumococcal polysaccharide vaccine (PPSV23) administered within six days of injury.

**Methods:**

This prospective observational study compared the immunogenicity of PPSV23 among critically ill burn and neurosurgical patients at a tertiary, academic medical center. Patients received PPSV23 vaccination within six days of ICU admission per standard of care. Consent was obtained to measure concentrations of vaccine-specific IgG to 14 of 23 serotype capsule-specific IgG in serum prior to and 14–35 days following PPSV23. A successful immunologic response was defined as both a ≥2-fold rise in capsule-specific IgG from baseline and concentrations of >1 mcg/mL to 10 of 14 measured vaccine serotypes. Immunologic response was compared between burn and neurosurgical patients. Multiple variable regression methods were used to explore associations of clinical and laboratory parameters to immunologic responses.

**Results:**

Among the 16 burn and 27 neurosurgical patients enrolled, 87.5% and 40.7% generated a successful response to the vaccine, respectively (p = 0.004). Both median post-PPSV23 IgG concentrations (7.79 [4.56–18.1] versus 2.93 [1.49–8.01] mcg/mL; p = 0.006) and fold rises (10.66 [7.44–14.56] versus 3.48 [1.13–6.59]; p<0.001) were significantly greater in burn compared with neurosurgical patients. Presence of burn injury was directly and days from injury to immunization were inversely correlated with successful immunologic response (both p<0.03). Burn injury was associated with both increased median antibody levels post-PPSV23 and fold rise to 14 vaccine serotypes (p<0.03), whereas absolute lymphocyte count was inversely correlated with median antibody concentrations (p = 0.034).

**Conclusion:**

Critically ill burn patients can generate successful responses to PPSV23 during acute injury whereas responses among neurosurgical patients is comparatively blunted. Further study is needed to elucidate the mechanisms of differential antigen responsiveness in these populations, including the role of acute stress responses, as well as the durability of these antibody responses.

## Introduction

Critically ill patients can exhibit profound inflammation and immunosuppression, and experience a high incidence of associated secondary infections [1]. Catastrophic injury and microbial invasion elicit an acute inflammatory response, often followed by a primary or compensatory anti-inflammatory response syndrome which can lead to an immunosuppressed state [1–6]. The presence of this proposed immunosuppressed state, which often accompanies sepsis, burn, trauma, and neurologic injury, is largely based on measurements of local and systemic immune parameters [2–8]. Such indirect measurements of immune function may not adequately assess the patient’s ability to generate an integrated response to specific antigenic challenge, such as vaccination.

Vaccines provide a relevant probe of immune integrity [9,10] and protection against secondary infections. The utility and interpretation of vaccine responsiveness in the diagnosis of immunodeficiency has been described by the American Academy of Allergy, Asthma and Immunology as well as a Joint Task Force on Practice Parameters last updated in 2015 [9,10]. As a screening tool for suspected primary immunodeficiency, specific antibody deficiency can be measured, in part, by IgG response to polysaccharide vaccines [10]. Immune function dynamics may vary between critically ill populations, and within a population due to individual patient variables. Specifically, critically ill burn patients display an immune dysregulation that often includes a down-regulation of immune signaling genes, decreased circulating dendritic cells, decreased monocyte human leukocyte antigen-DR, and derangements in cytokine excretion [6, 11–15]. Similarly, the immune consequences of catastrophic neurologic injury seen in ischemic and hemorrhagic stroke may be related to an impaired responsiveness of monocytes, lymphopenia, a cholinergic neuro-linked immune reflex, as well as activation of innate receptor signaling, e.g., by damage-associated molecular pattern pathways [16–21]. Thus, immune dysregulation following severe burn and stroke may contribute to the associated excessive morbidity and mortality [11, 12, 15, 16, 18] and affect the integrity of vaccine responses.

The Center for Disease Control and Prevention’s Advisory Committee on Immunization Practices recommends pneumococcal vaccination for all adults over 65 years of age and to those 19–64 years old with a high-risk condition for invasive pneumococcal disease [22]. Previously, the Joint Commission and the Centers for Medicare and Medicaid Services recommended core measure standards for accredited hospitals to improve coverage by administering pneumococcal vaccines to indicated patients prior to discharge [23]. Thus, our institution implemented an interdisciplinary 23-valent pneumococcal polysaccharide vaccine (PPSV23) protocol to encourage administration as early as possible, including to critically ill patients [24]. To determine whether systemic insults (e.g., burn or neurologic trauma) affected vaccine outcomes, we assessed the frequency and magnitude of pneumococcal serotype-specific antibody responses, and, in a subset of subjects, functional opsonophagocytic responses [25] to PPSV23 when administered within six days of initial insult, and associated clinical variables that may have affected these outcomes.

## Methods

The aim of this prospective observational cohort study was to comparatively evaluate the immunogenicity of PPSV23 in critically-ill burn and neurosurgical patients. We assessed patient variables associated with immunogenicity. In addition, multiplexed opsonophagocytosis assays (MOPA) were conducted as a feasibility pilot to assess antibody functionality. This study was approved by the Colorado Multiple Institution Review Board (13–2435) and conducted in accordance with board policies including prospective consent of participant or proxy.

### Patient enrollment

We prospectively enrolled 46 patients admitted to a burn or neurosurgical intensive care unit (ICU) for emergent/severe burn or neurologic injuries. Inclusion criteria included an indication and active order for PPSV23, age 19–88 years, ability to provide consent (or a known and present proxy), an expected ICU stay of at least 14 days, and an offending insult that had occurred within 6 days of PPSV23 being ordered. Burn patients (n = 18) required a total body surface area burn (TBSA) of 10% or greater (primary chemical, electrical and frostbite injuries were not eligible). Neurosurgical patients (n = 28) required the initial insult to be either an ischemic stroke, subarachnoid hemorrhage (SAH), intracranial/intraparenchymal hemorrhage (ICH/IPH) or subdural hemorrhage (SDH). Exclusion criteria were refusal of PPSV23 administration, lack of consent to study, known immune deficiency (including previous organ or bone marrow transplantation, HIV infection, active cancer or previous treatment with chemotherapy, and asplenia), primary admission for a planned intervention (such as skin grafting or neurosurgical intervention) or known previous pneumococcal vaccination. Written informed consent was obtained from the patient or their legal proxy if deemed appropriate by both the attending physician and nurse with protocols approved by the Colorado Multiple Institution Review Board.

### Immunization and samples

Patients were immunized intramuscularly in the left deltoid with 0.5 mL PPSV23 (Merck & Co., Inc.; Whitehouse Station, NJ), or in the right deltoid as required to avoid the location of burn injury. Ten mL of blood were collected in late afternoon prior to vaccine administration and 14–35 days following vaccination in serum separator tubes (BD; Franklin Lakes, NJ) from an existing intravenous line. Serum separator tubes were inverted a minimum of five times, allowed to clot in a vertical position for 30 minutes, and then centrifuged at 1300g for 20 minutes. Serum was removed and stored at -70° C until analyzed.

### Laboratory analyses

Prior to PPSV23 administration, we measured levels of C-reactive protein (CRP) by immunoturbidmetry and cortisol by chemiluminescent immunoassay in the clinical laboratory of the University of Colorado Hospital. Levels of pneumococcal capsule-specific IgG to 14 pneumococcal serotypes (1, 3, 4, 5, 6B, 7F, 8, 9N, 9V, 12F, 14, 18C, 19F and 23F) were tested by quantitative multi-analyte fluorescent detection method (Luminex, Luminex Corporation, Austin, TX) by ARUP laboratories (Salt Lake City, UT) with appropriate controls [26, 27]. As reported by ARUP, the lower limit of detection is 0.01 mcg/mL with inhibition by homologous serotypes of more than 95% (except serotypes 9V and 9N) and inhibition by heterologous serotypes of < 15% for all 14 measured serotypes. Correlation coefficients (r^2^) compared to ELISA was >0.85 for tested serotypes [27]. Intra-assay and inter-assay precision were reported at <20% and <30% coefficient of variation, respectively. Patients were informed of their specific IgG results and encouraged to discuss with their primary physician.

To characterize the function of capsule-specific IgG, a subset of samples (31 total, 13 matched pre-post vaccination and 5 post only) were analyzed by multiplexed opsonophagocytosis assay (MOPA) for serotypes 1, 3, 5, 7F, 6B, 14 and 23F at the University of Alabama-Birmingham, as described [25]. Opsonic indices (OIs) by MOPA analysis were defined as the reciprocal of the interpolated serum dilution that kills 50% of the pneumococcal serotype. Samples that did not kill 50% of the bacteria at the lowest dilution tested were reported as four, which was one-half the lowest dilution tested. Antibody potency (AP) was calculated by OI / IgG concentration (mcg/mL) [28]. Log transformed IgG concentration vs OI and OI vs AP were graphically inspected and analyzed for correlations.

### Definitions and statistical methods

We defined capsule-specific IgG responses to PPSV23 in serum as a composite outcome that reflects both a rise from baseline and absolute IgG concentration as previously recommended [9, 29–31]. We defined a successful composite immunologic response for each subject as the combination of a ≥2-fold rise in capsule specific IgG from baseline and levels > 1 mcg/mL for 10 of 14 serotypes. We also analyzed the proportion of subjects with 1) IgG concentrations of >1.3 mcg/mL for 10 of 14 serotypes following vaccination [9, 10, 32], or 2) fold rise from baseline of >4 for 10 of 14 serotypes [9, 31, 33]. These additional immunologic outcomes of “convalescent” IgG concentrations and “fold” rise from baseline, respectively, were utilized to assess agreement of interpretation based on alternative immunologic response definitions previously reported in the literature [9, 31–34]. A *post-hoc* analysis of immunologic response was conducted by additional criteria to assess agreement as defined by Moberley, et al [35] and Bonilla, et al [10]. Responsiveness was defined as absence of specific antibody deficiency (mild, moderate, or severe) using the 14 measured serotypes as well as seven serotypes (1, 3, 5, 8, 9N, 7F, 12F) not included in the previous 7-valent pneumococcal conjugate vaccine (PCV-7) [10].

Immunologic response was compared between burn and neurosurgical critically ill patients. Exploratory associations between clinical variables and immunogenicity were conducted in the whole critically ill population (both burn and neurosurgical combined). Categorical variables were analyzed by Fisher’s exact test. Continuous variables are reported as median with corresponding interquartile range [(IQR)] or mean with corresponding standard deviation (SD). T-test and Wilcoxon rank sum were utilized for comparisons between groups of normally distributed and non-parametric data, respectively. We identified univariate associations at a p-value < 0.2 between clinical and laboratory variables and successful composite immunologic responses. A multivariable logistic model was created from these significant variables by forward-step methods. Variables associated with patient-specific median post-PPSV23 IgG concentrations and median fold rise from baseline across all tested serotypes were assessed by forward-step linear regression using a model entry criteria of p < 0.2. All tests were two-sided with p-values < 0.05 considered significant and 95% confidence intervals (95% CI) reported.

## Results

Among 46 critically ill patients (18 burn and 28 neurosurgical patients) enrolled, three did not complete the study due to early discharge in two (burn patients) and one death (neurosurgical patient) before post-vaccination day 14. No severe adverse events related to PPSV23 were reported. Fever developed within 72 hours of PPSV23 administration in three (19%) burn and four (15%) neurosurgical patients. Six of the seven episodes were accompanied by culture positive infections (three respiratory, one blood, one urine, one skin). Clinically, the neurosurgical patients were older and more severely ill than those with burns, with higher APACHE scores and higher frequencies of the need for vasopressors and mechanical ventilation (Table 1). In contrast, laboratory markers of inflammation (numbers of white cells and CRP) were higher among burn patients.

**Table 1:** Patient demographics.

Characteristic	Burn (n = 16)	Neurosurgical (n = 27)	P-value
Gender (male), n (%)	10 (62.5)	13 (48.1)	0.53
Age, mean ± SD	40 ± 12	55.3 ± 13	<0.001
Ethnicity, n (%)			
• Hispanic	3 (18.8)	(22.2)	—
• Caucasian	13 (81.2)	66.7)	
• Black	0	(7.4)	
• Other	0	1 (3.7)	
Primary Diagnosis, n (%)			
	TBSA (mean ± SD): 23.9 ± 12.8	SAH: 19 (70)	—
		ICH: 3 (11)	
		SDH: 1 (4)	
		Ischemic: 4 (15)	
APACHE II, mean score ± SD	11.9 ± 6.4	17.8 ± 7.2	0.01
ICU length of stay, median days [IQR]	25 [13–30]	24 [17–30]	0.9
Required vasopressors, n (%)	5 (31.3)	20 (74.1)	0.01
Required mechanical ventilation, n (%)	10 (62.5)	21 (77.8)	0.28
Days from admission to PPSV23 administration, mean ± SD	2.9±1.9	3.9 ± 2.2	0.13
Time from vaccination to follow-up blood draw, median days [IQR]	14 [14–14]	14 [14–14]	0.99
White blood cell count^a^ (10^3^ cells/mcL), mean ± SD	20.5 ± 8.7	14.6 ± 3.4	0.02
Total lymphocytes^a^ (10^3^ cells/mcL), mean ± SD	1.8 ± 1.19	1.3 ± 0.65	0.16
Cortisol^a^ (mcg/dL), mean ± SD	13.7 ± 7	17 ± 9.5	0.22
CRP^a^ (mcg/dL), mean ± SD	16.2 ± 7.6	9.07 ± 6.8	0.005

TBSA, total burn surface area percent; SD, standard deviation; SAH, subarachnoid hemorrhage; ICH, intracerebral hemorrhage; SDH, subdural hemorrhage; PPSV23, 23-valent pneumococcal polysaccharide vaccine; IQR, interquartile range; CRP, C-reactive protein

a, values day of PPSV23 administration

**Fig 1:**
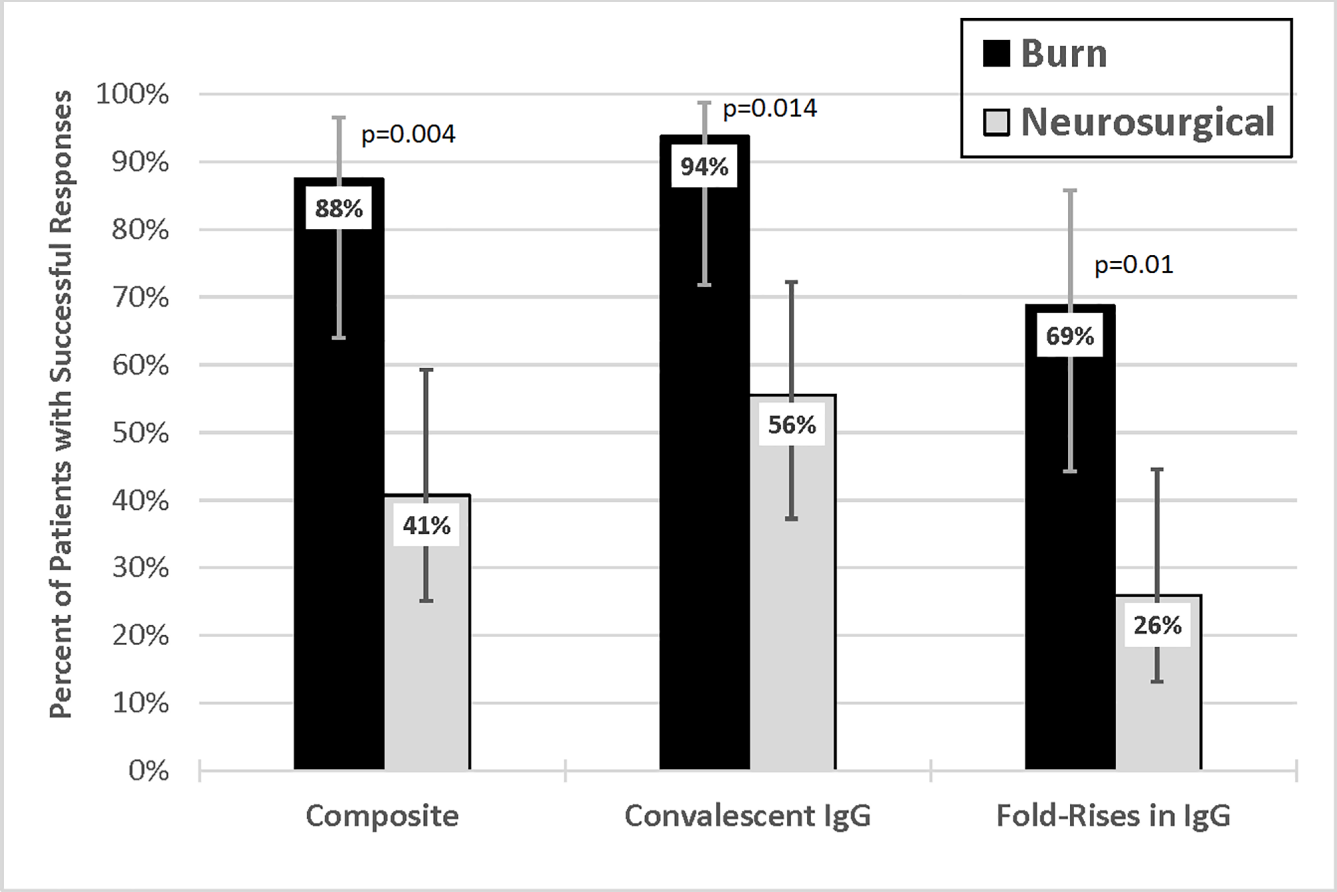
Proportion of patients in the ICU with burn and neurologic injuries who achieved successful responses to 23-valent pneumococcal polysaccharide vaccine. Proportion of response following 23-valent pneumococcal polysaccharide vaccine (PPSV23) by corresponding definition. Composite Response Outcome: IgG concentration ≥ 1 mcg/mL and 2 fold increase from baseline (successful immunologic response). Convalescent IgG Response Outcome: Post-vaccination IgG concentration of ≥ 1.3 mcg/mL. Fold-Rise Outcome: ≥ 4 fold increase from baseline. These definitions must be met by 10 of 14 measured serotypes to be considered a responder. Error bars indicate 95% confidence interval. IgG, Immunoglobulin concentration as microgram (mcg) per milliliter (mL).

Successful composite immunologic response frequencies were significantly higher among patients with burns than among those with neurologic injuries (88% vs. 41%; p = 0.004) (Fig 1). This pattern of immunologic response was consistent when analyzed by the proportion of subjects achieving an absolute “convalescent” concentration of capsule-specific IgG (94% vs 56%; p = 0.014) and fold rises (69% vs 26%; p = 0.01) in burn and neurosurgical patients after vaccination, respectively. This was consistent among *post-hoc* assessments of immunologic response favoring burn patients. Burn patients had an 81–94% response rate compared to a 33–41% response rate in neurosurgical patients depending on the *post-hoc* definition used (p<0.005 for all comparisons). There was relative agreement among outcomes when the composite immunologic response and all three *post-hoc* definitions were compared with three or less patients (<7%) being discordantly classified as responders or non-responders. All data is available in Table A in S1 Table. Dataset PPSV23 in Critically-ill.

In addition to the increased frequencies of immunologic success, patients admitted with burns also generated higher levels of capsule-specific IgG to 7 of 14 individual serotypes tested (Table 2). Moreover, burns were also accompanied by significantly increased fold-rises to 9 of 14 serotypes following immunization, despite similar baseline prevaccination values. Responses to serotypes 3, 4, 6B, 8, 14, 18C and 23F were most often increased during burn injury. Six of these 7 serotypes (all but type 8) are also included in the current 13-valent pneumococcal conjugate vaccine (PCV-13; Prizer Vaccines) and its antecedent PCV-7, serotypes that are most prominent in infants.

**Table 2:** Burn versus neurosurgical comparisons of serotype specific IgG concentrations pre and post vaccination and fold increase.

Serotype	Patient Population	Pre PPSV23 (mcg/mL)		P-value	Post PPSV23 (mcg/mL)		P-value	Fold Increase (Post/Pre)		P-value
1	Burn	0.38	[0.19–0.74]	0.38	6.06	[1.38–22.8]	0.156	14.31	[5.5–42.28]	0.077
	Neurosurgical	0.71	[0.18–1.03]		2.32	[1.2–7.69]		6.38	[1–24]	
3	Burn	0.54	[0.17–0.86]	0.97	2.37	[1.33–7.8]	***0*.*006***	7.03	[2.84–12.8]	***0*.*002***
	Neurosurgical	0.46	[0.2–0.9]		0.77	[0.41–2.4]		1.83	[1.25–4.75]	
4	Burn	0.25	[0.08–0.36]	0.44	2.69	[1.03–8.04]	***0*.*001***	21	[9.45–26.1]	***<0*.*001***
	Neurosurgical	0.24	[0.14–0.39]		0.59	[0.3–1.66]		1.88	[0.97–7.96]	
5	Burn	6	[3.56–8.88]	0.91	16.7	[9.06–31.09]	0.135	3.8	[1.51–6.39]	0.051
	Neurosurgical	6.9	[1.67–11.5]		10.7	[4.75–23.08]		1.39	[0.86–4.13]	
6B	Burn	0.48	[0.29–0.95]	0.16	7.32	[3.53–18.53]	***0*.*025***	13.85	[6.51–39]	***<0*.*001***
	Neurosurgical	0.85	[0.43–1.77]		2.33	[1–6.97]		1.92	[0.91–8.08]	
7F	Burn	0.99	[0.63–1.99]	0.51	6.94	[3.54–27.15]	0.085	9.93	[3.91–14.9]	***0*.*011***
	Neurosurgical	1.23	[0.65–3.52]		4.27	[1.71–12.13]		1.61	[0.99–11.1]	
8	Burn	0.75	[0.36–0.87]	0.61	9.81	[4.52–17.88]	***0*.*006***	13.01	[5.8–27.52]	***0*.*006***
	Neurosurgical	0.95	[0.49–1.61]		4.04	[1.31–6.8]		2.62	[0.98–10.9]	
9N	Burn	0.2	[0.16–0.76]	0.44	2.49	[1.49–7.45]	0.285	10.07	[4.48–23.8]	0.063
	Neurosurgical	0.37	[0.16–1.15]		1.75	[0.56–6.15]		3.56	[1.2–11.25]	
9V	Burn	0.65	[0.2–1.1]	0.17	12	[3.49–25.7]	0.145	18.99	[12.92–25]	***0*.*003***
	Neurosurgical	0.8	[0.38–1.76]		5.47	[1.71–16.37]		5.95	[1.3–15.55]	
12F	Burn	0.65	[0.33–0.99]	0.45	1.11	[1.02–19.65]	0.443	3.46	[1.8–14.97]	0.209
	Neurosurgical	0.33	[0.14–1.24]		1.39	[0.66–2.26]		2.17	[1.07–8.61]	
14	Burn	0.53	[0.36–1.25]	***0*.*014***	24.8	[3.96–48.2]	***0*.*037***	33.2	[12.4–64.4]	***<0*.*001***
	Neurosurgical	1.47	[0.65–5.29]		8.22	[2.15–16.7]		2.09	[0.95–14.7]	
18C	Burn	3	[2.55–4.96]	0.67	32.8	[10.54–43.7]	***0*.*004***	10.72	[5.58–16.7]	***0*.*003***
	Neurosurgical	2.46	[1–5.99]		6.88	[3.39–26.4]		3.35	[1.09–7.28]	
19F	Burn	2.75	[1.72–5.23]	0.8	19.9	[5.68–39.15]	0.061	4.16	[2.58–13.1]	0.085
	Neurosurgical	3.41	[1.08–5.3]		7.92	[2.71–17.3]		2.98	[1–4.77]	
23F	Burn	0.53	[0.25–1.94]	0.71	17.5	[3.71–18.35]	***0*.*002***	12.86	[7.49–36.9]	***0*.*001***
	Neurosurgical	0.62	[0.26–1.58]		2.64	[0.67–8.4]		2.52	[0.95–13.1]	

All values reported as median [interquartile range]; IgG, immunoglobulin; PPSV23, 23-valent pneumococcal polysaccharide vaccine

Variables associated with achieving immunologic success (p < 0.2) were considered for multivariable logistic regression by forward-step modeling (Table 3). A comparison of characteristics between patients achieving composite immunologic response and non-response is reported in Table 4. Clinically, burn diagnosis directly was significant independent positive predictor of immunologic success (Unit Odds 8.98, 95% CI 1.54–52.3, p = 0.015) as was days between hospital admission a negative predictor of vaccine success (0.67, 95% CI 0.47–0.96, p = 0.022), with the area under to response operator characteristic 0.82 (for both together).

**Table 3:** Associations of successful composite immunologic response with clinical and laboratory variables.

Variable	Univariate p-value	Forward step model		
		Coefficient estimate (95% CI)	p-value	Unit Odds (95% CI)
Burn diagnosis	0.004	1.09 (0.22–1.98)	0.015	8.98 (1.54–52.3)
Days from admission to PPSV23 administration	0.01	-0.4 (-0.76–-0.04)	0.022	0.67 (0.47–0.96)
Age	0.02	Age, CRP, WBC, Hospital non-survivor, and APACHE II score did not meet criteria to be significantly additive in association with the outcome of successful composite immunologic response by forward step method (p >0.2 for each).		
CRP	0.1			
Highest WBC within 24 hours of admission	0.12			
Hospital non-survivor	0.13			
APACHE II score	0.15			

PPSV23, 23-valent pneumococcal polysaccharide vaccine; CRP, C-reactive protein; WBC, white blood cell count; APACHE II, Acute Physiology and Chronic Health Evaluation II

**Table 4:** Comparison of patient characteristics by composite immunologic response.

Variable/Characteristic	Responder (n = 25)	Non-Responder (n = 18)	p-value
Injury type			—
• Burn, n (%)	14 (56)	2 (11)	0.004
• Neurologic, n (%)	11 (44)	16 (89)	
Gender (male), n (%)	14 (56)	9 (50)	0.99
Age, mean ± SD	45 ± 13.8	56 ±14.1	0.02
Days from admission to PPSV23 administration, mean ± SD	2.76 ± 1.67	4.5 ± 2.3	0.01
APACHE II, mean score ± SD	14.2 ± 6.5	17.6 ± 8.2	0.15
Required vasopressors, n (%)	13 (52)	12 (67)	0.37
Required mechanical ventilation, n (%)	18 (72)	13 (72)	0.99
Highest white blood cell count within 24 hours of admission (10^3^ cells/mcL), mean ± SD	18.2 ± 7.8	14.8 ± 3.4	0.12
White blood cell count at time of PPSV23 administration (10^3^ cells/mcL), mean ± SD	11.2 ± 6.8	12.3 ± 5	0.55
Total lymphocytes at time of PPSV23 administration (10^3^ cells/mcL), mean ± SD	1.33 ± 0.7	1.3 ± 0.6	0.87
Cortisol at time of PPSV23 administration (mcg/dL), mean ± SD	16 ± 8.5	15.5 ± 9.3	0.86
CRP at time of PPSV23 administration (mcg/dL), mean ± SD	13.3 ± 7.9	9.5 ± 7.4	0.1
Intensive care unit LOS, median [IQR]	25 [17.5–29.5]	22.5 [16.75–33.25]	0.7
Hospital LOS, median [IQR]	27[19–32]	30.5 [20.25–49]	0.54
Hospital non-survivor, n (%)	3 (12)	6 (33)	0.13

SD, standard deviation; PPSV23, 23-valent pneumococcal polysaccharide vaccine; CRP, C-reactive protein; LOS, length of stay; IQR, interquartile range

Forward-step multivariable linear regression modeling independently associated burn diagnosis with median post-PPSV23 IgG concentrations across all measured serotypes; whereas the absolute lymphocyte count closest to PPSV23 administration was inversely associated with median post-vaccine IgG concentrations. Days between hospital admission and vaccination, requiring vasopressors, age and APACHE II values were not statistically significant predictors of median post-PPSV23 IgG concentrations (Table 5). Only burn diagnosis was statistically predictive of median fold rise from baseline.

**Table 5:** Multivariable forward-step linear regression model for median post-PPSV23 IgG concentration and IgG fold-rise from baseline.

Median post-PPSV23 IgG concentration				Fold-rise in IgG from baseline				
Variable	Univariate	Multivariable regression		Variable		Univariate	Multivariable regression	
	p-value	Coefficient (95% CI)	p-value			p-value	Coefficient (95% CI)	p-value
**Burn diagnosis**	**0.006**	**2.29 (0.37–4.22)**	**0.02**	**Burn diagnosis**		**<0.001**	**3.08 (1.4–4.75)**	**<0.001**
**Absolute lymphocyte count closest to PPSV23 administration**	0.09	**-3.13 (-6–-0.24)**	**0.034**	CRP		0.031	NA	>0.2
Days from admission to PPSV23 administration	**0.041**	-0.74 (-1.63–0.15)	0.1	APACHE II score		0.04	NA	>0.2
Required vasopressors	0.052	NA	>0.2	Required vasopressors		0.1	NA	>0.2
Age	0.1	NA	>0.2	Highest WBC within 24 hours of admission		0.15	NA	>0.2
APACHE II score	0.14	NA	>0.2		

PPSV23, 23-valent pneumococcal polysaccharide vaccine; CRP, C-reactive protein; NA, not applicable since the variable was not included in the final model; APACHE II, Acute Physiology and Chronic Health Evaluation II

**Fig 2:**
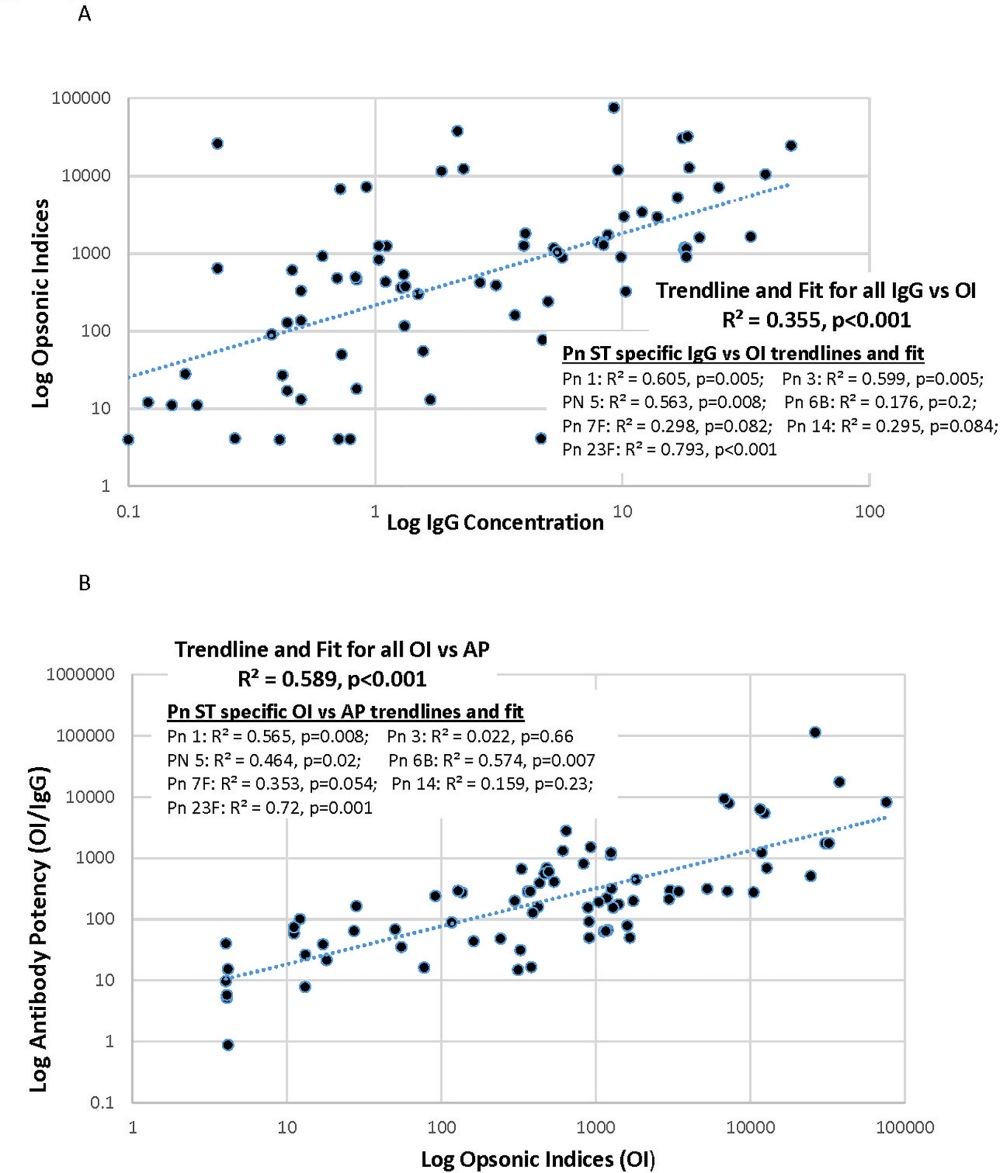
**A) Log-transformed pneumococcal serotype-specific IgG vs Opsonic index, B) Log-transformed pneumococcal serotype-specific Opsonic index vs antibody potency.** A, Log transformed weak association of pneumococcal serotype (Pn ST) specific immunoglobulin (IgG) concentrations and opsonic indices. B, Log transformed moderate association of pneumococcal serotype (Pn ST) specific opsonic indices and antibody potency.

Multiplexed opsonophagocytosis assay (MOPA) results were confounded by the clinical use of antibiotics in 20 of 31 planned samples. We report only results from 11 (4 obtained pre- and 7 post-vaccination) antibiotic negative samples tested against 7 serotypes. All data is available in Table B in S1 Table. Dataset PPSV23 in Critically-ill. The concentrations of vaccine capsule-specific IgG did appear to predict antibody function by MOPA. We found a consistent correlation between specific levels and the ability of sera to opsonize the related organisms overall (Opsonic Index; OI) and for 6 of 7 individual serotypes tested, particularly serotypes 1, 3, 5, and 23F (Fig 2A). Moreover, these OI’s also showed a significant correlation with the Antibody Potency, which reflects the opsonic activity per microgram of capsule-specific antibody (Fig 2B). Specific values are reported in Table 6. Thus, in this subset of critically ill patients, the antibodies produced in response to pneumococcal vaccination appear to demonstrate the functional ability to opsonize the relevant pneumococcal organisms, suggesting their potential to clear and kill these pathogens.

**Table 6:** Pneumococcal antibody opsonic indicies and antibody potency in serum of three burn and eight neurosurgical serum samples.

Capsular Serotype	Patient Population ^a^	Median OI	Range OI	Median Antibody Potency (OI/IgG)	Range Antibody Potency (OI/IgG)
1	Burn Post-PPSV23	1292	496–1748	200	154–598
	Neurosurgical Post-PPSV23	230	13–1657	47	8–200
	Neurosurgical Pre-PPSV23	8	4–11	32	5–74
3	Burn Post-PPSV23	391	241–464	127	48–552
	Neurosurgical Post-PPSV23	1033	431–1264	190	50–392
	Neurosurgical Pre-PPSV23	73	28–116	127	35–240
5	Burn Post-PPSV23	1046	313–7109	191	15–290
	Neurosurgical Post-PPSV23	201	13–381	21	16–31
	Neurosurgical Pre-PPSV23	16	4–50	40	1–69
6B	Burn Post-PPSV23	3427	901–6764	285	91–9395
	Neurosurgical Post-PPSV23	2419	1245–26302	786	298–114357
	Neurosurgical Pre-PPSV23	77	12–329	186	39–658
7F	Burn Post-PPSV23	12358	11855–12779	1236	686–5444
	Neurosurgical Post-PPSV23	15848	923–76009	1632	63–8244
	Neurosurgical Pre-PPSV23	766	361–7245	1540	156–7875
14	Burn Post-PPSV23	11551	10491–24596	510	277–6244
	Neurosurgical Post-PPSV23	3427	1402–37826	245	78–17594
	Neurosurgical Pre-PPSV23	722	481–1254	1013	687–1326
23F	Burn Post-PPSV23	1191	536–32242	412	67–1757
	Neurosurgical Post-PPSV23	798	4–2968	187	10–223
	Neurosurgical Pre-PPSV23	73	4–376	163	21–291

PPSV23, 23-valent pneumococcal polysaccharide vaccine; OI, Opsonic index (reciprocal of the interpolated serum dilution that kills 50% of the bacteria). For reporting purposes, serum that killed less than 50% of the bacteria are reported as 4, which is one half the lowest dilution of the serum tested. IgG, Immunoglobulin concentration as microgram per milliliter.

a, Three burn and four neurosurgical post PPSV23 samples and four neurosurgical pre PPSV23 samples were able to be analyzed due to the presence of anti-pneumococcal antibiotics being detected in the remaining samples

## Discussion

Critically ill patients may experience an immunosuppressed state independent of or following an inflammatory response to a physiologic insult [1–8, 16–18, 36, 37] and are at increased risk for acute infections. We prospectively tested the ability of two distinct critically ill populations in the ICU to respond to primary immunization (PPSV23) against a common cause of pneumonia, *S*. *pneumoniae* soon after admission. We found that critically ill burn patients frequently responded successfully to PPSV23. In contrast, critically ill neurosurgical patients had a blunted response to PPSV23 when compared with burn patients. Burn injury, as opposed to neurologic injury, was a common positive predictor of immunologic response in all multivariable analyses conducted. To our knowledge, immunologic response to PPSV23, as measured by pneumococcal serotype specific IgG, has not been investigated in these populations.

The differences in both the frequencies and the magnitudes of these humoral responses in these two populations may derive from differences in the severity of their illnesses and related immune parameters. Critically ill burn patients are stressed, hyperinflammatory and exhibit altered immune function compared with healthy controls, but their robust response to vaccine antigens compared with the relative infrequency of response in the neurosurgical patients deserves further exploration. Immune-dysfunction with brain injury and in neurosurgical critically ill patients also has been reported [7, 16–18]. Recent investigations suggest that immune modulation occurs in the acute and subacute phase of brain injury following ischemic stroke [18]. This maturation to an “exhausted immune phenotype” is consistent with a compensatory anti-inflammatory response framework [3, 8, 18]. Although our population largely included hemorrhagic injury as opposed to ischemic stroke, the inverse relationship between hospital day of PPSV23 administration and immunologic response also suggests a transition from inflammatory to immune-depressed phenotype in these patients. Whether differences in differential injury-related immune dysfunction in burn and neurosurgical insults as may be revealed by analyses of biomarkers, cytokines, pro-inflammatory and pro-resolution molecules underlie the striking differences in both the frequency and magnitude of acute immunogenicity are important areas for investigation.

In considering potential mechanisms and correlations of the differential responses to vaccine in these two populations, univariate associations were identified for age, APACHE II score, CRP, hospital mortality, WBC count. However, these clinical and laboratory variables were not independently predictive of immunologic success, whereas burn diagnosis and, inversely, admission day of PPSV23 administration were. Previous work has shown even advanced age does not preclude IgG response following PPSV23 compared to younger adults [38–41]. Amongst the independent associations identified by linear regression models for median concentrations of IgG post-PPSV23 administration and fold-rise from baseline, burn diagnosis was the common predictor of a more successful immunologic response. Based on these results, administration of PPSV23 in critically ill burn patients is reasonable. Other determinants should be considered to optimize outcomes among neurosurgical patients admitted with severe ischemic stroke or hemorrhagic conditions, such as timing of vaccine administration as responses were limited when given within six days of injury. Vaccine administration may need to be withheld until the patient is stabilized for discharge in order to elicit adequate immunogenicity. The role of the conjugated pneumococcal vaccine (PCV-13) to illicit immunogenicity remains unknown in critically ill patients, but should be considered for future study.

Logistically, we chose an immunologic success definition that incorporated both IgG value and fold rise. This outcome aligned well with other alternative definitions (absolute IgG concentrations >1.3mcg/mL or >4 fold rise from baseline), tools advanced for diagnosis of immune deficiencies and with establish laboratory standards [9, 10, 30–35]. As our primary definition was not the same as all others [10, 35], we assessed response and agreement using multiple definitions providing support to our overall interpretation. Further, we utilized 14 of 23 serotypes with a multiplex platform to represent immunogenicity. This test performance at ARUP compared to ELISA has been published [26, 27, 29, 30, 42], nevertheless should be considered when comparing to other methods as differences in performance may exist [32, 43]. Use of clinical diagnostic criteria may mitigate some risk of discordant characterization but caution is warranted when comparing results of different methods [9, 10, 26, 29, 32, 42, 43]. Testing of sera for vaccine responses predominantly at day 14 post-PPSV23 may have precluded identifying delayed peak responses to the vaccine over the following weeks. However, given the critical nature of our patient population, early sampling was implemented to avoid dropout due to death or transfer to outside facilities upon stabilization. Future studies will compare the pace of immune response in these potentially compromised populations.

Measuring pneumococcal sub-serotype IgG concentrations does not imply protection from future invasive illness. Results with selected samples showed a consistent correlation between capsule-specific IgG and opsonophagocytosis, a critical mechanism for clearing pneumococcal infections but we caution against over interpretation. Multiple retrospective and case-control studies confirm the protection afforded by PPSV23 against invasive pneumococcal disease [39] but a direct correlation with specific antibody levels (or “protective levels”) and function has not been established in any context [44, 45]. However, the potential impact of ineffective vaccination due to hyporesponsiveness in the neurologically compromised and elderly patients could result in increased rates of invasive pneumococcal disease in this at risk population. Although nosocomial pneumococcal infections occur, the primary goal of vaccination is durable protection in those at high risk [45]. Early pneumonia, of which pneumococcus is a common pathogen, following severe neurologic injury is well established and unlikely to benefit from immediate vaccination as preventative antibiotic therapy failed to prevent pneumonia [46,47]. Similarly in burn patients with a 0.5–3% incidence, pneumococcal infections tend to occur within the first week of injury [48,49]. Therefore, until additional data are known, vaccination upon stabilization from critical illness should be considered. Of note, our sample size limits our ability to draw firm conclusions about safety of PPSV23 in these populations even though no significant events were noted. However, it is comparable in size to other vaccine responsiveness studies in other populations such as recovered trauma patients who required splenic artery embolization or solid organ transplant recipients [50, 51].

In summary, a statistically significant difference in PPSV23 immunogenicity was found between critically ill burn and neurosurgical patients. To our knowledge this is the first observational study to assess immunogenicity of PPSV23 early after injury in critically ill burn and neurosurgical patients. Our findings suggest early administration of PPSV23 in the neurosurgical population fails to produce a robust immunologic response.

## Conclusion

Critically ill burn patients had a robust short term immunologic response following PPSV23 administration compared to the significantly blunted responses among critically ill neurosurgical patients. Further research into dysregulated immune pathways resulting in antigen non-responsiveness in neurosurgical patients is needed. The role of acute stress responses, optimal timing of PPSV23 administration, as well as the durability of immunogenicity in these distinct populations should be explored. Although, PPSV23 appears safe in these critically ill patients, a differential immunologic response based on underlying insult may occur.

## Supporting information

S1 Table Dataset PPSV23 in critically-ill. Inclusive data supporting analyses of immunologic response (Table A) and multiplexed opsonophagocytosis assay output of interest (Table B).
(PDF)

## References

[1] van Vught LA, Klein Klouwenberg PMC, Spitoni C, Scicluna BP, Wiewel MA, Horn J, et al Incidence, risk factors, and attributable mortality of secondary infections in the intensive care unit after admission for sepsis. JAMA. 2016;315(14):1469–1479. doi: 10.1001/jama.2016.2691 26975785 10.1001/jama.2016.2691

[2] Lord JM, Midwinter MJ, Chen Y, Belli A, Brohl K, Kovacs EJ, et al The systemic immune response to trauma: an overview of pathophysiology and treatment. Lancet. 2014;384:1455–65. doi: 10.1016/S0140-6736(14)60687-5 25390327 10.1016/S0140-6736(14)60687-5 PMC4729362

[3] Boomer JS, To K, Chang KC, Takasu O, Osborne DF, Walton AH, et al Immunosuppression in patients who die of sepsis and multiple organ failure. JAMA. 2011;306:2594–2605. doi: 10.1001/jama.2011.1829 22187279 10.1001/jama.2011.1829 PMC3361243

[4] Namas RA, Vodovotz Y, Almahmoud K, Abdul-Malak O, Zaaqoq A, Namas R, et al Temporal patterns of circulating inflammation biomarker networks differentiate susceptibility to nosocomial infection following blunt trauma in humans. Ann Surg. 2016;263:191–198. doi: 10.1097/SLA.0000000000001001 25371118 10.1097/SLA.0000000000001001 PMC5136774

[5] Wong HR, Lindsell CJ, Pettila V, Meyer NJ, Thair SA, Karlsson S, et al A Multibiomarker-based outcome risk stratification model for adult septic shock. Crit Care Med. 2014;42:781–789. doi: 10.1097/CCM.0000000000000106 24335447 10.1097/CCM.0000000000000106 PMC4620515

[6] Mace JE, Park MS, Mora AG, Chung KK, Martini W, White CE, et al Differential expression of the immunoinflammatory response in trauma patients: burn vs. non-burn. Burns. 2012;38:599–606. doi: 10.1016/j.burns.2011.10.013 22103986 10.1016/j.burns.2011.10.013 PMC9479166

[7] Courties G, Moskowitz MA, Nahrendorf M. The innate immune system after ischemic injury: lessons to be learned from the heart and brain. JAMA Neurol. 2014;71:233–236. doi: 10.1001/jamaneurol.2013.5026 24296962 10.1001/jamaneurol.2013.5026 PMC3946050

[8] Ward NS, Casserly B, Ayala A. The compensatory anti-inflammatory response syndrome (CARS) in critically ill patients. Clin Chest Med. 2008;39:617–vii. 10.1016/j.ccm.2008.06.010 PMC2786900 18954697

[9] Orange JS, Ballow M, Stiehm ER, Ballas ZK, Chinen J, De La Morena M, et al Use and interpretation of diagnostic vaccination in primary immunodeficiency: a working group report of the basic and clinical immunology interest section of the American Academy of Allergy, Asthma and Immunology. J Allergy Clin Immunol 2012;130:S1–24. doi: 10.1016/j.jaci.2012.07.002 22935624 10.1016/j.jaci.2012.07.002

[10] Bonilla FA, Khan DA, Ballas ZK, Chinen J, Frank MM, Hsu JT, et al Practice parameter for the diagnosis and management of primary immunodeficiency. J Allergy Clin Immunol 2015;136:1186–1205 (e1-78). doi: 10.1016/j.jaci.2015.04.049 26371839 10.1016/j.jaci.2015.04.049

[11] Xiu F, Jeschke M. Perturbed mononuclear phagocyte system in severely burned and septic patients. Shock. 2013;40:81–88. doi: 10.1097/SHK.0b013e318299f774 23860581 10.1097/SHK.0b013e318299f774 PMC3734943

[12] Venet F, Tissot S, Debard AL, Faudot C, Crampe C, Pachot A, et al Decreased monocyte human leukocyte antigen-DR expression after severe burn injury: correlation with severity and secondary septic shock. Crit Care Med. 2007;35:1910–1917. doi: 10.1097/01.CCM.0000275271.77350.B6 17568330 10.1097/01.CCM.0000275271.77350.B6

[13] D’Arpa N, Accardo-Palumbo A, Amato G, D’Amelio L, Pileri D, Cataldo V, et al Circulating dendritic cells following burns. Burns. 2009;35:513–518. doi: 10.1016/j.burns.2008.05.027 19269101 10.1016/j.burns.2008.05.027

[14] Moore CB, Medina MA, van Deventer HW, O’Connor BP, Cameron S, Taxman DJ, et al Downregulation of immune signaling genes in patients with large surface burn injury. J Burn Care Res. 2007;28:879–887. doi: 10.1097/BCR.0b013e318159a41e 17925653 10.1097/BCR.0b013e318159a41e

[15] Davis CS, Albright JM, Carter SR, Ramirez L, Kim H, Gamelli RL, Kovacs EJ. Early pulmonary immune hyporesponsiveness is associated with mortality after burn and smoke inhalation injury. J Burn Care Res. 2012;33:26–35. doi: 10.1097/BCR.0b013e318234d903 21979852 10.1097/BCR.0b013e318234d903 PMC3253958

[16] Pavlov VA, Tracey KJ. The vagus nerve and the inflammatory reflex-linking immunity and metabolism. Nat Rev Endocrinol. 2012;8:743–754. doi: 10.1038/nrendo.2012.189 23169440 10.1038/nrendo.2012.189 PMC4082307

[17] Olofsson PS, Rosas-Ballina M, Levine YA, Tracey KJ. Rethinking inflammation: neural circuits in the regulation of immunity. Immunological Reviews. 2012;248:188–204. doi: 10.1111/j.1600-065X.2012.01138.x 22725962 10.1111/j.1600-065X.2012.01138.x PMC4536565

[18] Liesz A, Dalpke A, Mracsko E, Antoine DJ, Roth S, Zhou W, et al DAMP signaling is a key pathway inducing immune modulation after brain injury. J Neurosci. 2105;35(2):583–598. 10.1523/JNEUROSCI.2439-14.2015 PMC4293412 25589753

[19] Przybycien-Szymanska MM, Ashley WW. Biomarker discovery in cerebral vasospasm after aneurysmal subarachnoid hemorrhage. Journal of Stroke and Cerebrovascular Diseases. 2015;24:1453–1464. doi: 10.1016/j.jstrokecerebrovasdis.2015.03.047 25957908 10.1016/j.jstrokecerebrovasdis.2015.03.047

[20] Badjatia N, Carpenter A, Fernandez L, Schmidt JM, Mayer SA, Claassen J, et al Relationship between c-reactive protein, systemic oxygen consumption, and delayed cerebral ischemia after aneurysmal subarachnoid hemorrhage. Stroke. 2011;42:2436–2442. doi: 10.1161/STROKEAHA.111.614685 21757662 10.1161/STROKEAHA.111.614685

[21] Tapia-Perez JH, Karagianis D, Zilke R, Koufuglou V, Bondar I, Schneider T. Assessment of systemic cellular inflammatory response after spontaneous intracerebral hemorrhage. Clinical Neurology and Neurosurgery 2016;150:72–79. doi: 10.1016/j.clineuro.2016.07.010 27611984 10.1016/j.clineuro.2016.07.010

[22] United States Department of Health and Human Services: Centers for Disease control and Prevention’s Advisory Committee on Immunization Practices. Recommended Adult Immunization Schedule: 2016. Available at: http://www.cdc.gov/vaccines/schedules/downloads/adult/adult-combined-schedule.pdf. Last accessed 9/23/2016.

[23] The Joint Commission. Specifications Manual for National Hospital Inpatient Quality Measures. Available at http://www.jointcommission.org/core_measure_sets.aspx. Last accessed 8/30/2016.

[24] Wall GC, Van Der Veer JJ, Romine MJ, Yeager SM. Assessment of candidacy for pneumococcal vaccination in intensive care patients. Intensive Crit Care Nurs 2013;29:212–215. doi: 10.1016/j.iccn.2012.10.004 23201039 10.1016/j.iccn.2012.10.004

[25] Song JY, Moseley MA, Burton RL, Nahm MH. Pneumococcal vaccine and opsonic pneumococcal antibody. J Infect Chemother 2013;19(3)412–425. doi: 10.1007/s10156-013-0601-1 23657429 10.1007/s10156-013-0601-1 PMC3692352

[26] Pickering JW, Larson MT, Martins TB, Copple SS, Hill HR. Eliminating of false-positive results in a luminex assay for pneumococcal antibodies. Clinical and Vaccine Immunology 2010;17:185–189. doi: 10.1128/CVI.00329-09 19923569 10.1128/CVI.00329-09 PMC2812081

[27] Pickering JW, Martins TB, Greer RW, Schroder MC, Astill ME, Litwin CM, et al A multiplexed fluorescent microsphere immunoassay for antibodies to pneumococcal capsular polysaccharides. Am J Clin Pathol 2002;117:589–596. doi: 10.1309/4KEH-AGY7-UT5H-41XJ 11939734 10.1309/lmch-c4q2-vfl9-3t1a

[28] Nahm MH and Burton RL. Protocol for multiplexed opsonophagocytic killing assay (UAB-MOPA) for antibodies against Streptococcus pneumoniae. Version E.02, December 2014. Available at: http://www.vaccine.uab.edu/UAB-MOPA.pdf. Last accessed January 27, 2017

[29] Daly TM, Pickering JW, Zhang X, Prince HE, Hill HR. Multilaboratory assessment of threshold versus fold-change algorithms for minimizing analytical variability in multiplexed pneumococcal IgG measurements. Clin Vaccine Immunol. 2014;21(7):982–8. doi: 10.1128/CVI.00235-14 24807051 10.1128/CVI.00235-14 PMC4097436

[30] Daly TM, Hill HR. Use and Clinical Interpretation of Pneumococcal Antibody Measurements in the Evaluation of Humoral Immune Function. Clin Vaccine Immunol. 2015;22(2):148–152. doi: 10.1128/CVI.00735-14 25520149 10.1128/CVI.00735-14 PMC4308869

[31] Hare ND, Smith BJ, Ballas ZK. Antibody response to pneumococcal vaccination as a function of preimmunization titer. J Allergy Clin Immunol 2009;123:195–200. doi: 10.1016/j.jaci.2008.09.021 18951616 10.1016/j.jaci.2008.09.021 PMC3613280

[32] Zhang X, Simmerman K, Yen-Lieberman B, Daly TM. Impact of analytical variability on clinical interpretation of multiplex pneumococcal serology assays. Clinical and Vaccine Immunology 2013;20(7):957–61. doi: 10.1128/CVI.00223-13 23677324 10.1128/CVI.00223-13 PMC3697459

[33] ARUP Laboratories. Interpretive information: streptococcus pneumoniae antibodies, IgG (14 serotypes). Available at: ltd.aruplab.com/Tests/Pub/0050725. Accessed February 15, 2017.

[34] Borgers H, Meyts I, De Boeck K, Raes M, Sauer K, Proesmans M, et al Fold-increase in antibody titer upon vaccination with pneumococcal unconjugated polysaccharide vaccine. Clin Immunol 2012;145(2):136–8. doi: 10.1016/j.clim.2012.08.010 23026475 10.1016/j.clim.2012.08.010

[35] Moberley S, Licciardi PV, Balloch A, Andrews R, Leach AJ, Kirkwood M, et al Repeat pneumococcal polysaccharide vaccine in Indigenous Australian adults is associated with decreased immune responsiveness. Vaccine 2017;35:2908–2915. doi: 10.1016/j.vaccine.2017.04.040 28455171 10.1016/j.vaccine.2017.04.040

[36] Thompson CM, Park CH, Maier RV, O’Keefe GE. Traumatic injury, early gene expression, and gram-negative bacteremia. Crit Care Med 2014;42:1397–1405. doi: 10.1097/CCM.0000000000000218 24561564 10.1097/CCM.0000000000000218 PMC4515113

[37] Hotchkiss RS, Monneret G, Payen D. Immunosuppression in sepsis: a novel understanding of the disorder and a new therapeutic approach. Lancet Infect Dis 2013;13:260–68. doi: 10.1016/S1473-3099(13)70001-X 23427891 10.1016/S1473-3099(13)70001-X PMC3798159

[38] Musher DM, Groover JE, Graviss EA, Baughn RE. The lack of association between aging and postvaccination levels of IgG antibody to capsular polysaccharides of Streptococcus pneumonia. Clin Infect Dis. 1996;22:165–7. 8824989 10.1093/clinids/22.1.165

[39] Carson PJ, Nichol KL, O’Brien J, Hilo P, Janoff EN. Immune function and vaccine responses in healthy advanced elderly patients. Arch Intern Med. 2000;160:2017–2024. 10888975 10.1001/archinte.160.13.2017

[40] Lee H, Nahm MH, Kim KH. The effect of age on the response to the pneumococcal polysaccharide vaccine. BMC Infectious Disease. 2010;10:60. 10.1186/1471-2334-10-60 PMC2856571 20219110

[41] Serpa JA, Valayam J, Musher DM, Rossen RD, Pirofski L, Rodriguez-Barradas MC. Vh3 antibody response to immunization with pneumococcal polysaccharide vaccine in middle-aged and elderly persons. Clin Vaccine Immunol. 2011;18:362–366. doi: 10.1128/CVI.00408-10 21228144 10.1128/CVI.00408-10 PMC3067391

[42] Hill HR, Pickering JW. Reference laboratory agreement on multianalyte pneumococcal antibody results: an absolute must! Clin Vaccine Immunol 2013;20:955–956. doi: 10.1128/CVI.00325-13 23697576 10.1128/CVI.00325-13 PMC3697438

[43] Balloch A, Licciardi PV, Tang MLK. Serotype-specific anti-pneumococcal IgG and immune competence: critical differences in interpretation criteria when different methods are used. J Clin Immunol 2013;33:335–341. doi: 10.1007/s10875-012-9806-9 23054341 10.1007/s10875-012-9806-9

[44] Moberley S, Holden J, Tatham DP, Andrews RM. Vaccines for preventing pneumococcal infection in adults. Cochrane Database Syst Rev 2013;1: doi: 10.1002/14651858.CD000422.pub3 23440780 10.1002/14651858.CD000422.pub3 PMC7045867

[45] Janoff EN, Musher DM: *Streptococcus pneumonia* In: Mandell, Douglas, and Bennett’s Principles and Practice of Infectious Diseases. Updated Eighth Edition Bennett JE, Dolin R, Blaser MJ (Eds). Philadelphia, PA, Elsevier, 2015, p. 2310–2327.

[46] Westendorp WF, Vermeij JD, Zock E, Hooijenga IJ, Kruyt ND, Bosboom HJLW, et al The preventative antibiotics in stroke study (PASS): a pragmatic randomized open-label masked endpoint clinical trial. Lancet 2015;385:1519–1526. doi: 10.1016/S0140-6736(14)62456-9 25612858 10.1016/S0140-6736(14)62456-9

[47] Vermeij JD, Westendorp WF, Dippel DW, van de Beek D, Nederkoorn PJ. Antibiotic therapy for preventing infections in people with acute stroke. Cochrane Database Syst Rev 2018;1:CD008530 doi: 10.1002/14651858.CD008530.pub3 29355906 10.1002/14651858.CD008530.pub3 PMC6491314

[48] Glasser JS, Landrum ML, Chung KK, Hospenthal DR, Renz EM, Wolf SE, et al Description of streptococcus pneumonia infections in burn patients. Burns 2010;36(4):528–32. doi: 10.1016/j.burns.2009.07.006 19765906 10.1016/j.burns.2009.07.006

[49] Costa Santos D, Barros F, Gomes N, Guedes T, Maia M. Face and/or neck burns: a risk factor for respiratory infections? Ann Burns Fire Disasters 2016;29(2):97–102. 28149229 PMC5286993

[50] Olthof DC, Lammers AJJ, van Leeuwen EMM, Hoekstra JB, ten Berge IJ, Goslings JC. Antibody response to a T-cell-independent antigen is preserved after splenic artery embolization for trauma. Clin Vaccine Immunol 2014;21(11):1500–4. doi: 10.1128/CVI.00536-14 25185578 10.1128/CVI.00536-14 PMC4248772

[51] Eckerle I, Rosenberger KD, Zwahlen M, Junghanss T. Serologic vaccination response after solid organ transplantation: a systematic review. PLoS ONE 2013;8(2): e56974 doi: 10.1371/journal.pone.0056974 23451126 10.1371/journal.pone.0056974 PMC3579937

