# Supplementary material for: Divergent humoral responses to 23-valent pneumococcal polysaccharide vaccine in critically-ill burn and neurosurgical patients
Source: PLoS One. 2018 May 14;13(5):e0197037. doi: 10.1371/journal.pone.0197037 (PMC5951595; doi:10.1371/journal.pone.0197037)
Supplement: S1 Table — Inclusive data supporting analyses of immunologic response (Table A) and multiplexed opsonophagocytosis assay output of interest (Table B). (PDF) [file pone.0197037.s001.pdf]

S1 Table. Dataset PPSV23 in Critically-ill

Table A: Clinical and IgG Concentrations

De-identified subject	Group (Burn = 1, Neuro = 0)	Pre ST 1	Pre ST 3	Pre ST 4	Pre ST 5	Pre ST 8	Pre ST 6B	Pre ST 9N	Pre ST 7F	Pre ST 12F	Pre ST 14	Pre ST 19F	Pre ST 9V	Pre ST 23F	Pre ST 18C
1	1	0.12	0.18	0.26	3.75	0.33	0.42	0.21	0.45	0.45	0.39	3.06	0.56	0.12	2.71
2	1	0.11	0.77	0.18	7.87	0.26	0.94	0.18	12.81	1.12	3.17	0.59	0.18	7.46	4.72
3	1	2.77	0.78	0.4	6.17	0.84	1.45	0.27	3.74	0.84	3.27	4.59	0.72	0.87	3.12
4	1	0.7	0.56	0.19	2.67	0.74	0.42	0.19	0.93	0.33	0.44	1.1	0.93	0.31	2.55
5	1	0.36	3.29	0.36	6.33	0.82	0.87	1.27	0.69	1.36	0.42	8.15	1.12	0.62	6.52
6	1	0.2	1.81	0.07	17.44	2.11	0.66	0.14	1.16	0.54	0.38	0.66	1.25	0.09	2.53
7	1	0.14	0.09	0.31	11.11	18.59	0.27	0.09	0.46	0.96	0.3	2.4	0.17	0.05	0.88
8	1	0.86	0.55	0.28	5.91	0.78	1.16	0.71	1.97	1.07	0.74	1.93	1.1	2.15	8.37
9	1	0.07	0.1	0.04	1.05	0.37	0.1	0.09	0.95	0.14	0.65	6.08	0.29	1.87	0.87
10	1	0.45	1.09	0.36	18.55	7.27	1.91	1.23	0.73	0.96	5.28	14.79	1.44	2.27	2.88
11	1	0.39	0.52	0.23	4.53	0.57	0.45	0.44	1.02	0.75	0.61	4.95	0.58	3.23	5.67
12	1	0.25	0.2	0.05	3.18	0.27	0.14	0.13	2.08	0.31	0.22	0.81	0.2	0.11	2.58
13	1	0.48	0.39	0.36	8.13	0.75	0.51	0.89	1.24	0.24	1.69	3.98	0.71	0.44	6.53
14	1	0.2	0.08	0.06	3.68	0.21	0.29	0.17	0.2	0.44	0.28	2.43	0.19	0.3	0.46
15	1	1.07	0.14	0.08	2.75	0.65	0.2	0.17	0.28	0.17	0.29	2.08	0.11	0.29	3.74
16	1	3.08	4.59	0.42	11.55	0.97	0.99	2.86	2.14	1.21	1.1	6.47	1.18	1.61	3.89
1	0	0.46	0.44	0.21	36.38	1.23	0.85	0.24	24.89	2.57	2.01	5.5	1.26	0.49	4.96
2	0	0.12	0.46	0.24	26.01	0.19	6.75	2.4	1.01	1.46	0.22	1.82	1.74	5.08	12.05
3	0	1.23	0.93	0.3	25.17	1.74	0.88	0.49	4.35	1.84	12.99	12.67	14.29	1.07	9.29
4	0	0.83	0.25	0.78	6.27	0.53	0.42	0.14	0.9	0.87	2.77	3.52	1.34	0.12	1.62
5	0	0.94	0.91	0.58	10.41	1.01	1.51	0.85	0.75	1.23	1.47	3.89	0.56	0.8	3.24
6	0	1.19	0.69	0.37	7.76	3.18	1.53	0.79	6.37	1.14	1.2	5.59	0.8	2.37	0.98
7	0	1.15	0.54	0.2	3.86	1.27	0.37	0.84	1.23	0.18	3.69	3.06	1.59	2.12	1.53
8	0	0.1	0.19	0.06	3.84	0.47	0.15	0.18	0.18	0.16	0.42	4.48	0.18	0.29	1.64
9	0	1.03	0.26	0.24	3.98	0.38	0.44	0.37	6.62	2.02	0.41	3.49	0.3	0.19	1.31
10	0	1.54	0.1	0.13	10.82	0.58	7.23	0.09	0.62	0.01	1.52	1.29	0.34	0.72	3.11
11	0	2.8	0.54	0.51	12.2	2.93	3.51	5.09	2.24	0.28	4.8	5.75	13.73	7.8	10.02
12	0	1.03	0.21	0.68	12.28	10.52	2.51	3.77	0.68	0.43	5.58	3.07	1.78	1.83	3.74
13	0	0.79	1.56	0.34	4.68	1.49	0.5	0.92	1.27	0.3	0.46	6.62	0.52	0.84	5.62
15	0	0.73	0.16	0.71	9.9	3.18	2	1.38	4.62	0.35	34.2	1.91	1.01	0.53	4.92
16	0	1.02	8.92	0.4	7.17	1.73	1.17	6.34	1.53	1.29	10.79	6.94	12.68	14.16	8.04
21	0	0.12	0.55	0.3	13.37	0.81	1.31	0.18	2.69	0.33	0.34	2.14	0.71	0.33	28.52
22	0	0.12	0.15	0.29	27.05	1.42	0.14	7.81	2.55	4.05	9.81	5.13	4.13	0.87	6.17
24	0	0.53	0.16	0.14	10.42	0.18	0.97	0.15	0.21	0.01	0.66	3.59	0.95	0.22	0.41
25	0	0.24	0.24	0.1	1.88	0.6	0.64	0.2	0.37	0.09	0.64	0.68	0.16	0.2	1.81
27	0	1.23	0.89	0.76	6.9	7.91	7.81	4.82	13.37	0.81	14.68	16.2	7.24	3.61	15.75
29	0	0.05	2.04	0.23	0.36	0.25	0.14	0.08	0.4	0.07	0.54	0.36	0.42	0.16	2.46
30	0	0.17	1.33	0.11	1.45	0.5	4.92	0.36	1.09	1.25	5	0.47	0.73	0.13	1.03
31	0	0.38	0.16	0.18	1.23	0.61	0.26	0.17	1.83	0.12	0.78	0.84	0.15	0.62	0.62
40	0	0.19	0.17	0.06	0.42	0.18	0.12	0.07	5.71	0.1	1.03	0.21	0.23	0.1	0.66
41	0	0.15	0.38	0.07	0.27	0.13	0.5	0.11	0.23	0.18	1.03	0.55	0.11	0.44	0.49
42	0	0.56	0.51	0.15	0.77	0.95	0.76	0.41	0.38	0.19	8.7	0.86	4.77	0.43	0.62
43	0	0.71	1.31	0.1	0.73	1.26	0.44	0.1	0.92	0.08	0.7	3.41	0.56	1.32	0.16

S1 Table. Dataset PPSV23 in Critically-ill

Post ST 1	Post ST 3	Post ST 4	Post ST 5	Post ST 8	Post ST 6B	Post ST 9N	Post ST 7F	Post ST 12F	Post ST 14	Post ST 19F	Post ST 9V	Post ST 23F	Post ST 18C	Fold increase Sero Type 1	Fold increase Sero Type 3
1.34	2.27	7.31	9.9	12.44	20.02	7.7	3.27	1.03	19.44	59.27	12.98	18.35	47.5	11.17	12.61
0.83	1.56	0.71	31.33	4.26	4.6	2.22	28.27	2.31	48.2	1.5	0.77	9.06	39.78	7.55	2.03
6.61	5.97	2.87	42.77	19.13	37.83	3.48	5.45	1.03	48.2	23.07	13.54	18.35	8.98	2.39	7.65
5.51	0.69	1.77	24.51	9.67	5.09	6.68	1.43	0.72	1.85	3.45	6.65	18.35	9.51	7.87	1.23
0.99	18.13	8.18	8.91	19.13	36.42	9.71	6.04	23.65	30.21	59.27	28.74	18.35	42.03	2.75	5.51
1.48	2.47	0.34	21.17	4.56	6.65	0.61	28.27	0.91	4.62	18.6	10.94	1.38	30.12	7.4	1.36
0.68	1.24	3.11	42.77	18.34	0.72	0.15	9.59	1.11	1.42	12.48	3.24	1.3	24.82	4.86	13.78
32.71	8.82	11.97	5.43	8.22	35.31	1.31	28.27	7.62	48.2	41.65	35.53	18.35	44.29	38.03	16.04
2.5	0.84	0.99	9.51	4.5	7.98	4.88	7.83	1.08	50	16.36	23.48	17.88	9.88	35.71	8.4
28.56	3.08	7.61	50	9.94	14.06	9.6	4.13	50	50	31.66	26.2	16.2	28.99	63.47	2.83
23.15	1.48	2.26	8.2	16.48	0.64	2.13	3.39	1.16	37.94	7.37	7.97	17.77	12.51	59.36	2.85
28.03	1.28	1.13	14.6	15.4	2.18	2.01	50	0.68	16.4	2.38	2.53	1.08	36.94	112.12	6.4
8.37	5	9.54	30.38	50	3.17	23.44	18.62	1.11	50	57.6	33.33	17.29	50	17.44	12.82
8.74	8.41	0.66	17.57	2.35	5.77	2.75	2.27	50	3.21	21.91	4.22	1.93	5.55	43.7	105.13
21.67	1.7	2.5	5.47	8.42	12.01	1.07	3.98	1.02	3.74	5.11	2.08	50	50	20.25	12.14
4.78	13.26	8.76	15.6	4.09	9.88	2.02	23.77	50	18.21	21.24	24	16.72	35.53	1.55	2.89
1.35	0.69	0.19	17.08	11.14	1.06	0.23	28.27	2.5	48.2	11	18.51	18.35	46.55	2.93	1.57
10.12	1.09	8.09	30.79	0.38	6.97	23.87	1.71	1.39	1.98	7.92	33.49	18.35	40.41	84.33	2.37
1.2	1.03	0.46	21.71	4.04	0.83	0.56	4.32	1.76	12.32	16.12	16.69	1.12	10.55	0.98	1.11
3.08	0.47	6.21	9.87	2.08	1.25	0.29	2.8	1.08	48.2	59.27	24.52	0.21	4.4	3.71	1.88
7.69	1.46	11.97	36.27	6.33	37.83	13.75	7.1	23.65	3.47	9.94	4.79	17.69	45.36	8.18	1.6
1.43	0.69	0.36	5.42	5.84	3.83	1.4	4.27	1.09	1.06	4.07	1.83	1.64	2.23	1.2	1
16.17	5.92	3.47	31.38	19.13	16.44	23.87	28.27	13.92	48.2	40.92	15.28	14.49	47.5	14.06	10.96
0.55	0.25	0.09	4.56	16.61	0.65	1.87	0.6	0.33	10.36	17.3	5.47	0.86	9.77	5.5	1.32
0.77	0.19	0.19	2.95	0.31	0.37	0.29	4.06	2.26	0.32	2.71	0.23	0.16	1.07	0.75	0.73
1.52	0.16	0.18	12.85	0.62	6.55	0.16	1.71	0.14	1.38	1.64	0.48	0.67	3.39	0.99	1.6
2.32	0.68	0.62	15.99	2.87	3.11	6.15	2.71	0.3	8.22	5.73	16.37	8.4	9.84	0.83	1.26
1.19	0.41	0.61	14.57	6.8	3.55	3.86	0.67	0.61	7.87	3.22	2.05	2.64	5.59	1.16	1.95
33.02	18.13	0.64	23.12	19.13	4.04	23.87	17.47	1.96	16.7	19.72	8.07	13.85	37.19	41.8	11.62
0.71	0.19	0.49	6.89	19.13	2.33	1.66	4.06	0.76	33.57	1.79	1.55	0.35	4.56	0.97	1.19
24.44	13.63	1.66	29.62	8.43	18	23.87	24.03	4.66	45.64	32.95	16.49	13.44	37.51	23.96	1.53
3.79	1.26	0.3	23.08	1.4	1.1	1.71	3.09	1.57	0.22	1.96	1.01	0.31	26.4	31.58	2.29
0.12	0.12	0.27	21.84	1.33	0.12	8.38	2.37	3.41	9.25	4.08	3.92	0.85	5.58	1	0.8
19.14	0.76	0.69	42.77	3.75	8.23	0.84	15.14	1.34	3.46	15.08	14.24	2.91	6.64	36.11	4.75
1.53	0.56	0.68	10.69	1.41	2.26	2.25	5.5	1.46	7.15	3.59	1.3	2.14	6.88	6.38	2.33
1.67	3.99	1.04	10.32	6.33	10.14	5.88	17.79	1.49	20.49	16.76	13.49	5.27	18.5	1.36	4.48
3.67	18.13	7.73	0.5	5.83	1.11	1.97	0.61	0.11	8.01	7.78	10.15	2.65	17.9	73.4	8.89
4.08	1.66	0.59	5.81	1.31	1.67	1.28	12.13	3.41	8.37	2.24	35.53	4.92	2.45	24	1.25
3.69	0.77	0.35	1.23	6.63	1	0.34	1.09	0.71	1.31	28.75	6.64	1.56	2.72	9.71	4.81
1.49	1.1	0.49	4.75	1.1	0.23	0.74	9.22	0.66	2.15	0.64	1.12	0.41	2.39	7.84	6.47
4.29	4.56	1.87	8.94	4.37	25.52	1.86	4.71	1.55	16.5	27.94	1.71	5.77	4.6	28.6	12
0.3	0.31	0.05	0.2	0.53	0.21	0.11	0.12	0.07	4.06	0.27	2.4	0.1	0.18	0.54	0.61
14.46	2.4	0.31	3.68	1.21	4.43	1.75	4.34	1.38	10.31	12.39	3.33	4.18	7.25	20.366	1.8321

S1 Table. Dataset PPSV23 in Critically-ill

Fold increase Sero Type 4	Fold increase Sero Type 5	Fold increase Sero Type 8	Fold increase Sero Type 6B	Fold increase Sero Type 9N	Fold increase Sero Type 7F	Fold increase Sero Type 12F	Fold increase Sero Type 14	Fold increase Sero Type 19F	Fold increase Sero Type 9V	Fold increase Sero Type 23F	Fold increase Sero Type 18C	Primary: combined responsiv eness	>1mcg/mL serotype respons 14/14	>1mcg/mL Serotype response 10/14	>1mcg/mL Serotype response 8/14
28.12	2.64	37.7	47.67	36.67	7.27	2.29	49.85	19.37	23.18	152.92	17.53	1	1	1	1
3.94	3.98	16.38	4.89	12.33	2.21	2.06	15.21	2.54	4.28	1.21	8.43	1	0	1	1
7.18	6.93	22.77	26.09	12.89	1.46	1.23	14.74	5.03	18.81	21.09	2.88	1	1	1	1
9.32	9.18	13.07	12.12	35.16	1.54	2.18	4.2	3.14	7.15	59.19	3.73	1	0	1	1
22.72	1.41	23.33	41.86	7.65	8.75	17.39	71.93	7.27	25.66	29.6	6.45	1	0	1	1
4.86	1.21	2.16	10.08	4.36	24.37	1.69	12.16	28.18	8.75	15.33	11.91	0	0	1	1
10.03	3.85	0.99	2.67	1.67	20.85	1.16	4.73	5.2	19.06	26	28.2	0	0	1	1
42.75	0.92	10.54	30.44	1.85	14.35	7.12	65.14	21.58	32.3	8.53	5.29	1	1	1	1
24.75	9.06	12.16	79.8	54.22	8.24	7.71	50	2.69	80.97	9.56	11.36	1	0	1	1
21.14	50	1.37	7.36	7.8	5.66	50	50	2.14	18.19	7.14	10.07	1	1	1	1
9.83	1.81	28.91	1.42	4.84	3.32	1.55	62.2	1.49	13.74	5.5	2.21	1	0	1	1
22.6	4.59	57.04	15.57	15.46	50	2.19	74.55	2.94	12.65	9.82	14.32	1	0	1	1
26.5	3.74	50	6.22	26.34	15.02	4.63	99	14.47	46.94	39.3	50	1	1	1	1
11	4.77	11.19	19.9	16.18	11.35	99	11.46	9.02	22.21	6.43	12.07	1	0	1	1
31.25	1.99	12.95	60.05	6.29	14.21	6	12.9	2.46	18.91	50	50	1	1	1	1
20.86	1.35	4.22	9.98	0.71	11.11	99	16.55	3.28	20.34	10.39	9.13	1	1	1	1
0.9	0.47	9.06	1.25	0.96	1.14	0.97	23.98	2	14.69	37.45	9.39	0	0	1	1
33.71	1.18	2	1.03	9.95	1.69	0.95	9	4.35	19.25	3.61	3.35	1	0	1	1
1.53	0.86	2.32	0.94	1.14	0.99	0.96	0.95	1.27	1.17	1.05	1.14	0	0	1	1
7.96	1.57	3.92	2.98	2.07	3.11	1.24	17.4	16.84	18.3	1.75	2.72	0	0	1	1
20.64	3.48	6.27	25.05	16.18	9.47	19.23	2.36	2.56	8.55	22.11	14	1	1	1	1
0.97	0.7	1.84	2.5	1.77	0.67	0.96	0.88	0.73	2.29	0.69	2.28	0	0	1	1
17.35	8.13	15.06	44.43	28.42	22.98	77.33	13.06	13.37	9.61	6.83	31.05	1	1	1	1
1.5	1.19	35.34	4.33	10.39	3.33	2.06	24.67	3.86	30.39	2.97	5.96	0	0	0	0
0.79	0.74	0.82	0.84	0.78	0.61	1.12	0.78	0.78	0.77	0.84	0.82	0	0	0	0
1.38	1.19	1.07	0.91	1.78	2.76	14	0.91	1.27	1.41	0.93	1.09	0	0	0	0
1.22	1.31	0.98	0.89	1.21	1.21	1.07	1.71	1	1.19	1.08	0.98	0	0	1	1
0.9	1.19	0.65	1.41	1.02	0.99	1.42	1.41	1.05	1.15	1.44	1.49	0	0	1	1
1.88	4.94	12.84	8.08	25.95	13.76	6.53	36.3	2.98	15.52	16.49	6.62	1	0	1	1
0.69	0.7	6.02	1.17	1.2	0.88	2.17	0.98	0.94	1.53	0.66	0.93	0	0	0	1
4.15	4.13	4.87	15.38	3.76	15.71	3.61	4.23	4.75	1.3	0.95	4.67	1	1	1	1
1	1.73	1.73	0.84	9.5	1.15	4.76	0.65	0.92	1.42	0.94	0.93	0	0	1	1
0.93	0.81	0.94	0.86	1.07	0.93	0.84	0.94	0.8	0.95	0.98	0.9	0	0	0	1
4.93	4.1	20.83	8.48	5.6	72.1	134	5.24	4.2	14.99	13.23	16.2	1	0	1	1
6.8	5.69	2.35	3.53	11.25	14.86	16.22	11.17	5.28	8.13	10.7	3.8	1	0	1	1
1.37	1.5	0.8	1.3	1.22	1.33	1.84	1.4	1.03	1.86	1.46	1.17	0	1	1	1
33.61	1.39	23.32	7.93	24.63	1.53	1.57	14.83	21.61	24.17	16.56	7.28	1	0	1	1
5.36	4.01	2.62	0.34	3.56	11.13	2.73	1.67	4.77	48.67	37.85	2.38	1	0	1	1
1.94	1	10.87	3.85	2	0.6	5.92	1.68	34.23	44.27	2.52	4.39	0	0	0	1
8.17	11.31	6.11	1.92	10.57	1.61	6.6	2.09	3.05	4.87	4.1	3.62	0	0	0	1
26.71	33.11	33.62	51.04	16.91	20.48	8.61	16.02	50.8	15.55	13.11	9.39	1	1	1	1
0.33	0.26	0.56	0.28	0.27	0.32	0.37	0.47	0.31	0.5	0.23	0.29	0	0	0	0
3.1	5.041	0.96	10.07	17.5	4.717	17.25	14.73	3.633	5.946	3.1667	45.3125	1	0	1	1

S1 Table. Dataset PPSV23 in Critically-ill

>1.3mcg/mL Serotype response 14/14	>1.3mcg/mL Serotype response 10/14	>1.3mcg/mL Serotype response 8/14	>2 fold response 14/14	>2 fold response 10/14	>2 fold response 8/14	>4 fold response 14/14	>4 fold response 10/14	>4 fold response 8/14	Cortisol	CRP (mcg/dL · note units)	Percent Burn	Neuro Process (Isch = 1, SDH = 2, ICH/IPH = 3, SAH = 4)	Days from admission to vaccination
0	1	1	1	1	1	0	1	1	10.6	23.7	9		3
0	1	1	0	1	1	0	0	0	21	5.1	20		1
0	1	1	0	1	1	0	1	1	14	16	49		2
0	1	1	0	1	1	0	0	1	21	13.6	20		2
0	1	1	0	1	1	0	1	1	21	23.5	20		2
0	1	1	0	1	1	0	1	1	4	15.7	15		6
0	0	1	0	1	1	0	0	1	11	17.5	15		2
1	1	1	0	1	1	0	1	1	13.7	7	54		3
0	1	1	1	1	1	0	1	1	16.3	10.8	17		7
1	1	1	0	1	1	0	1	1	24	5	19		1
0	1	1	0	1	1	0	0	0	5	18.1	27		2
0	1	1	1	1	1	0	1	1	7	28.8	19		3
0	1	1	1	1	1	0	1	1	1	7.2	41		2
0	1	1	1	1	1	1	1	1	13.2	25.6	15		6
0	1	1	0	1	1	0	1	1	15	23.88	20		3
1	1	1	0	1	1	0	0	1	22	17.6	23		1
0	1	1	0	0	0	0	0	0	29	23.5		4	6
0	1	1	0	0	1	0	0	0	30	1.1		3	1
0	0	1	0	0	0	0	0	0	32	16.2		3	6
0	0	1	0	1	1	0	0	0	3	8		1	0
1	1	1	0	1	1	0	1	1	15	10.6		4	5
0	1	1	0	0	0	0	0	0	12	21.7		4	7
1	1	1	1	1	1	1	1	1	22	11.1		3	2
0	0	0	0	1	1	0	0	0	16	10		3	5
0	0	0	0	0	0	0	0	0	14.2	10.1		4	4
0	0	0	0	0	0	0	0	0	16	4.2		2	7
0	1	1	0	0	0	0	0	0	3	1.5		4	6
0	0	1	0	0	0	0	0	0	20	0.2		1	0
0	1	1	0	1	1	0	1	1	34	8.2		4	2
0	0	1	0	0	0	0	0	0	20	2.1		3	5
1	1	1	0	1	1	0	0	1	3	3.7		4	2
0	0	1	0	0	0	0	0	0	12	4.4		4	5
0	0	1	0	0	0	0	0	0	22	2.07		4	4
0	1	1	1	1	1	1	1	1	13	20.89		4	3
0	1	1	1	1	1	0	1	1	10	17.87		4	3
0	1	1	0	0	0	0	0	0	18.3	8.7		4	7
0	1	1	0	1	1	0	1	1	30	1.75		4	6
0	1	1	0	1	1	0	0	0	6	11.74		4	4
0	0	0	0	0	1	0	0	0	10	17.41		4	3
0	0	0	0	1	1	0	0	1	32	3.77		4	6
1	1	1	1	1	1	1	1	1	14	10.25		4	2
0	0	0	0	0	0	0	0	0	4	3.82		4	2
0	1	1	0	1	1	0	0	1	17	9.89		4	1

S1 Table. Dataset PPSV23 in Critically-ill

Days between vaccine and f/u blood draw	Age	Hospital LOS	ICU LOS	Median fold increase across all serotypes	WBC pre (highest w/in 24 hours admission)	Absolute Lymphocytes (admission)	Absolute Lymphocytes (closest to PPSV23)	WBC 24 hr Post	Required Pressors (Y = 1)	Gender (F=1)	ALL Median post IgG	APACHE II	Use of steroids (Y = 1)
19	50	9	9	21.273926	9.7	2.4	1.5	6.3	0	0	11.17	4	0
10	32	11	11	4.129359	16.1	2.5	3.8	9.3	0	0	3.285	13	0
14	19	77	77	7.4144231	19.9	2	0.5	1.5	1	0	11.26	20	0
14	35	30	30	7.5109831	18.3	5	1.4	5.2	0	0	5.3	14	0
14	33	17	17	13.071665	32.5	1	1.2	12.9	0	1	18.74	8	0
14	47	22	18	8.076	15.5	1.6	1.1	8.5	0	1	4.59	5	0
14	56	49	40	5.0285714	11.6	0.6	1	6.4	1	1	2.265	19	0
14	56	270	270	15.193309	29.7	0.5	0.3	5.1	1	1	23.31	20	0
14	40	30	25	11.759242	14.9	1.3	2.4	32	0	0	7.905	10	0
14	26	17	12	8.9354251	13.3	0.8	1.5	4.4	0	0	19.925	7	0
15	19	18	22	4.0822193	23.2	1.9	1.9	3.5	0	1	7.67	10	0
10	36	20	7	14.889684	30.9	1.1	1.1	18.9	0	0	2.455	8	0
15	49	29	25	21.887289	13.9	0.9	0.9	3	0	0	18.875	5	0
14	53	26	25	11.764752	14.9	1.7	1.4	11.4	0	0	4.885	6	0
14	45	31	26	13.584066	39.7	3.7	1.5	14.1	1	0	4.545	19	0
13	45	30	29	9.556737	23.7	1.5	1.3	6.6	1	1	16.16	23	0
14	36	50	48	1.78	20.2	2.2	1	22.5	1	1	11.07	26	0
14	59	83	29	3.4828658	13	1.7	1.7	16.1	1	1	8.005	17	1
14	75	33	21	1.0771279	12.8	1	1.1	10.4	0	0	2.9	25	0
15	31	91	13	3.0436508	16.7	0.9	0.8	16.7	1	0	2.94	23	0
14	75	33	29	9.010119	9.3	0.3	0.9	23.5	1	0	10.955	14	0
14	37	30	26	0.9864865	20.8	3.3	0.9	17.8	1	0	1.735	18	1
14	44	50	37	14.561931	14.3	0.3	1	13	0	0	17.785	19	0
14	52	41	26	4.0974702	11.3	0.5	0.6	8.1	1	0	1.365	3	0
14	55	34	27	0.7821358	15.1	1.3	1.3	14.8	1	0	0.345	17	0
15	56	49	49	1.2294667	16.1	1.4	0.9	9.7	1	0	1.025	26	0
14	69	17	17	1.1346014	16	1.8	2.4	18.3	0	1	4.42	14	1
14	82	22	2	1.1709109	8.7	1.7	1.7	8.7	0	0	2.93	15	0
14	55	23	19	12.230361	16.1	1	1.3	10.6	0	1	17.8	10	0
14	76	18	18	0.9770908	13	0.9	2	9.2	1	1	1.725	24	0
14	53	20	19	4.1899212	11.2	1.5	0.5	13.6	1	1	20.935	11	1
14	59	31	31	1.285617	14.8	1.3	1.1	11.4	1	1	1.485	15	0
14	51	21	17	0.9302231	13.1	0.9	1.8	7.6	1	0	2.89	7	0
14	50	27	18	10.855904	21.1	0.9	0.9	11.4	1	1	5.195	14	1
14	66	22	21	6.5875	16.6	1.2	0.9	11.2	1	0	2.195	27	0
14	43	56	57	1.3630723	14.1	1.2	1.2	15.8	0	0	8.235	18	0
14	42	56	56	15.697917	14.1	1	2	15.8	1	0	4.75	27	0
14	38	24	24	3.7812261	11.4	2.1	1.3	12.6	1	1	2.93	11	0
14	58	24	24	4.1166253	13	0.8	0.6	9.8	1	0	1.27	20	0
14	61	18	16	5.4903382	11.9	1.1	1.6	6	0	1	1.1	8	0
14	63	30	30	18.693676	11.3	1.2	1.1	8.1	1	1	4.58	20	0
14	64	8	8	0.3245614	21.3	1.5	2.3	19.4	1	1	0.205	34	0
14	42	14	14	5.4937622	16.6	2.3	1	10.2	1	1	3.93	17	1

S1 Table. Dataset PPSV23 in Critically-ill

Mechanical Ventilation (Yes = 1)	Hospital Mortality (Yes = 1, died this stay)	Change in WBC Pre - Post	Post hoc yes/no outcomes to include:	Response in 5 of 7 PPSV23 only ST > 1.3mcg = 1, MOD SAD = 0	Fold increase >2 fold in 5 of 7 PPSV23 only = 1, failure = 0	>1.3 and >2 fold increase in 5 0f 7 PPSV23 only = 1, Moderate or MILD SAD = 0	NOTE 1= YES response Moberley "OR" def (>50%)	NOTE: 1 = YES response, Moberley "OR" def (>70%)	1 = NO SAD, responders to 70% of all measured ST
0	0	3.4		1	1	1	1	1	1
1	0	6.8		1	1	1	1	1	1
1	0	18.4		1	1	1	1	1	1
1	0	13.1		1	1	1	1	1	1
1	0	19.6		1	1	1	1	1	1
0	0	7		1	0	0	1	1	1
1	0	5.2		0	0	0	1	0	0
1	0	24.6		1	1	1	1	1	1
1	0	-17.1		1	1	1	1	1	1
0	0	8.9		1	1	1	1	1	1
0	0	19.7		1	1	1	1	1	1
0	0	12		1	1	1	1	1	1
0	0	10.9		1	1	1	1	1	1
1	0	3.5		1	1	1	1	1	1
1	0	25.6		1	1	1	1	1	1
1	0	17.1		1	0	0	1	1	1
1	0	-2.3		1	0	0	1	0	0
1	0	-3.1		1	0	0	1	0	0
1	1	2.4		0	0	0	0	0	0
1	0	0		0	0	0	1	0	0
1	0	-14.2		1	1	1	1	1	1
1	1	3		1	0	0	0	0	0
1	1	1.3		1	1	1	1	1	1
1	0	3.2		0	1	0	0	0	0
0	1	0.3		0	0	0	0	0	0
1	0	6.4		0	0	0	0	0	0
1	0	-2.3		1	0	0	0	0	0
0	0	0		0	0	0	0	0	0
0	0	5.5		1	1	1	1	1	1
1	1	3.8		0	0	0	0	0	0
0	0	-2.4		1	1	1	1	1	1
1	1	3.4		1	0	0	0	0	0
0	0	5.5		1	0	0	0	0	0
1	0	9.7		1	1	1	1	1	1
1	1	5.4		1	1	1	1	1	1
1	0	-1.7		1	0	0	1	1	0
1	0	-1.7		0	0	0	1	1	1
1	0	-1.2		1	1	1	1	1	1
1	0	3.2		0	0	0	0	0	0
0	0	5.9		0	1	0	1	0	0
1	0	3.2		1	1	1	1	1	1
1	1	1.9		0	0	0	0	0	0
1	1	6.4		1	1	1	1	1	1

S1 Table. Dataset PPSV23 in Critically-ill

Table B: Opsonic Indices

Identifier	Pn 1 OI	Pn 3 OI	Pn 5 OI	Pn 6B OI	Pn 7F OI	Pn 14 OI	Pn 23F OI
N13 POST	1657	1159	381	1817	30571	5254	2968
N27 POST	13	1264	324	3020	1124	1599	1175
N29 POST	161	907	13	1245	923	1402	421
N40 POST	298	431	77	26302	76009	37826	4
N13 PRE	4	55	4	136	361	610	18
N40 PRE	11	28	27	12	888	833	4
N41 PRE	11	91	4	329	643	1254	128
N43 PRE	4	116	50	17	7245	481	376
B13 POST	1292	391	313	901	11855	10491	1191
B14 POST	1748	241	7109	3427	12779	24596	32242
B2 POST	496	464	1046	6764	12358	11551	536
